# The prevalence and effect of burnout on graduate healthcare students

**Published:** 2017-06-30

**Authors:** Garrett Bullock, Lynnea Kraft, Katherine Amsden, Whitney Gore, Bobby Prengle, Jeffrey Wimsatt, Leila Ledbetter, Kyle Covington, Adam Goode

**Affiliations:** 1 Doctor of Physical Therapy Program, Duke University, North Caroline, US

## Abstract

Burnout is a growing epidemic among professional healthcare students. Unaddressed burnout has been shown to have psychological and performance related detriments. The purpose of this scoping literature review was to investigate the prevalence of burnout and its effects on the psychological, professional, empathetic ability, and academic acuity of graduate healthcare students. Inclusion criteria included English language papers published within the last 10 years and subjects in graduate healthcare professional programs. This search encompassed 8,214 articles. After title and abstract screening, 127 articles remained and were sorted into five domains of interest: etiology, professionalism, mental health, empathy, and academic performance. After duplicates were removed, 27 articles remained for the scoping review. Graduate level healthcare students had higher levels of burnout than age matched peers and the general population. The high prevalence of burnout within graduate healthcare students can have an effect on their mental health, empathy, and professional conduct. Understanding the occurrence and effects of burnout within graduate healthcare programs allows faculty and administration to plan curriculum, and provide information to students to understand, recognize, and create opportunities to decrease burnout in order to create long lasting quality clinicians.

## Introduction

Stress is a common problem throughout graduate healthcare professional students’ education.^1^–^4^ It has been reported that the prevalence of higher stress levels in medical students can range from 31% to as high as 73%.^2^–^4^ One consequence of high and continuous levels of stress is burnout.^5^ Burnout is generally defined as emotional and physical exhaustion resulting from a combination of exposures to environmental and internal stressors as well as inadequate coping and adaptive skills.^6^ Previous authors^6^–^8^ have demonstrated that burnout rates are not only higher in medical students than in the general population, but also that the prevalence of burnout increases as graduate healthcare students, or those students pursing a doctoral healthcare degree†, progress through their respective programs.^5^–^7^ This increased prevalence of burnout, has been shown to affect academic performance, mental health, and quality of life of graduate healthcare students.^9^, ^10^

Previous work found an increase in prevalence of burnout in graduate healthcare students^5^–^7^ and subsequent negative effects on mental health, quality of life, and performance.^11^–^14^ The purpose of this scoping literature review was to rigorously investigate the prevalence of burnout and its effects on the psychological, professional, empathetic ability, and academic acuity of graduate healthcare students. We hypothesized that the literature would demonstrate that burnout decreases students’ abilities to cope with psychological stressors, perform professionally and empathetically, and achieve academic excellence.

## Methods

To study the hypothesis, a systematic and comprehensive scoping review was performed on the prevalence of burnout and the effects burnout has on graduate healthcare students.^15^ This scoping review was performed in order to understand the literature, identify research gaps, categorize study types, and recognize study populations. After an initial search, a total of five burnout domains were created: etiology, professionalism, mental health, empathy, and academic performance. The five domains were generated based upon the prevalence of at least two studies repeating the same theme. Furthermore, each domain was deemed relevant to how burnout could influence practice, policy-making, curriculum, and pedagogy within graduate healthcare students’ coursework.^15^

### Data source and search

A librarian-assisted computerized search was conducted in PubMed, ERIC, and CINAHL in May 2016. An updated search of PubMed, CINAHL, and ERIC were conducted in September 2016. Searches were completed, with an initial screening of PubMed, followed by investigations into the additional databases. Medical Subject Headings (MeSH) terms and selected free-text terms were utilized for burnout, academic performance, stress, anxiety, depression, mental health, resiliency, altruism, graduate students, health occupations students, and medical school (see Appendix A for detailed search). The bibliographies of included literature were also hand searched for missing publications. Citations were tracked in EndNote (version X7, Thomas Reuters).

### Inclusion/exclusion criteria

Articles found in the literary search were examined to meet the guidelines as set by the following inclusion and exclusion criteria:

- Inclusion Criteria
  ○ Papers written from January 2006 through December 2015.
  ○ Studies examining burnout and its effects on professionalism (based on the healthcare code of ethics or standardized tools), mental health, empathy, or academic performance in graduate healthcare students.
  ○ English language papers, regardless of country of origin.
- Exclusion Criteria
  ○ Papers that included any students who were not specified to be doctoral degree-seeking students in a healthcare professional program beyond a bachelor’s degree.
  ○ Papers that focused on practicing professionals, unless they were only used as a reference measure.

The study population requirements included students at the graduate level in the healthcare profession such as medical, dental, and physical therapy students. Studies were included that discussed factors related to burnout. These factors included the three focus areas of burnout (emotional exhaustion, depersonalization, and low sense of personal accomplishment),^16^ suicidal ideation, professional behavior, quality of life, depression, resiliency, and empathy.^6^,^10^,^11^,^17^,^18^

In reviewing title and abstracts, we identified the previously referenced domains: etiology, mental health, professionalism, empathy, and academic acuity. These themes were subsequently divided among individual reviewers. The reviewers then collected articles according to their theme and completed full-text reviews. A second reviewer further examined articles to confirm or deny study viability. In case of disagreement, a third reviewer was incorporated.

### Data abstraction

Data were abstracted into a customized Excel spreadsheet by separate investigators for each individual burnout domain. A different investigator verified data for each domain. Disagreements concerning data were resolved by a third investigator’s opinion. Data elements included study characteristics (e.g., publication date, population, exposures, outcome measures utilized), results (prevalence within populations, outcomes), conflict of interest, and author’s conclusions.

### Data synthesis

The abstracted data were aggregated into the five previously referenced domains. The domains entailed the prevalence of burnout on graduate healthcare students, the effect of burnout on altruistic behavior, professional conduct, academic performance, and mental health. These domains were then summarized to describe populations, exposure, definition of burnout, outcome definition, and timing, and key findings.

## Results

**Figure 1:**
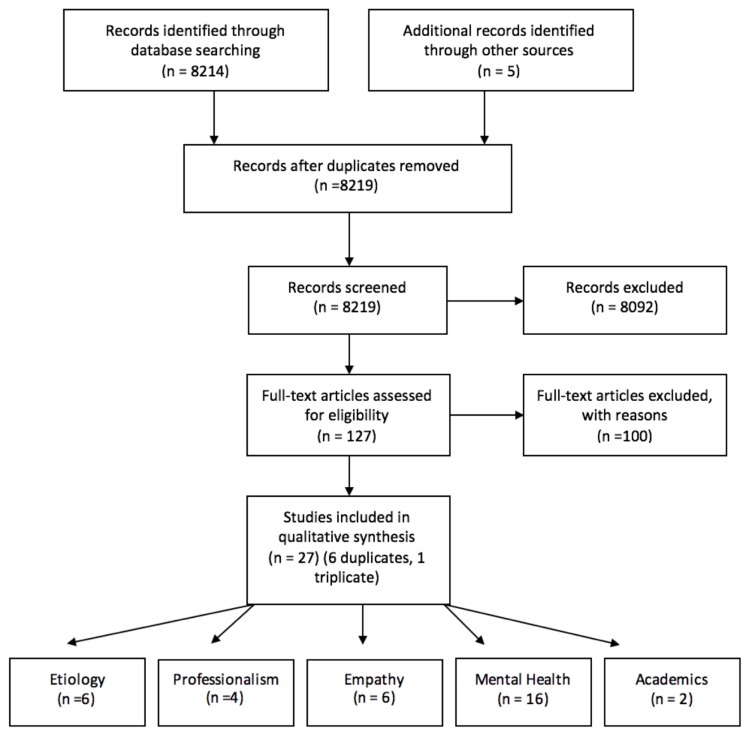
Prisma Flow Diagram

Citations identified via the search amounted to 8,214. An additional five articles were found from hand searching citations that met the search criteria. After screening for eligibility, 4,255 citations remained for title review. The titles were screened by two reviewers for applicability to burnout and its implications on graduate healthcare students. This left 187 articles for abstract review. Articles with themes not replicated in at least one other article were also removed (n =60). Based on theme, the 127 citations were divided among the five reviewers for full-text review. Following full-text review, 22 articles were hand-searched for additional resources. After duplicates were removed, 27 articles remained for the scoping review. The review process can be seen in Figure 1. Further explanation of groups created from these 27 articles can be seen in tables 1–5 (Appendix B).

### Etiology

The demographics and stress-related causes among medical school students were analyzed to determine their correlation with burnout (Table 1 in Appendix B). Data were obtained via online surveys and screenings. Five of the six studies utilized the Maslach Burnout Inventory Human Services Survey (MBI-HSS). All except Dyrbye et al.,^10^ which compiled data based on a search that used the Medical Subject Heading terms: medical student and depression, professional burnout, mental health, depersonalization, distress, anxiety, or emotional exhaustion. Twenty-one to fifty five percent of studies reported a moderate to high degree of burnout in medical students. Lapinski et al.^19^ and Chang et al.^20^ reported a significant negative correlation between burnout in medical students and level of support provided on campus, by family, or friends. Students who scored greater than 35 on the stress scale were more than two times as likely to experience burnout. Two separate studies yielded results with conflicting results: Lapinski et al.^19^ found that females were 1.5 times more likely to experience burnout in comparison to males, while Dyrbye et al.^18^ determined no significant difference between genders for being at risk for burnout. Overall, the majority of studies found that less support, increased stress, and progression to later years in medical school are strongly correlated with burnout.

### Professionalism

Detailed study characteristics for professionalism are presented in Table 2 (Appendix B). All studies incorporated a cross-sectional cohort design through survey response. All four trials surveyed graduate medical school students, with cohorts ranging in size from 127 to 2682 (61%-71.8% response rate) subjects. The Maslach Burnout Inventory (MBI), with the three areas of focus (emotional exhaustion, depression, and depersonalization) was administered in all four trials. Dyrbye^11^ and Dyrbye et al.^22^ included personal belief questions based on the Medical Students’ Attitudes toward Providing Care for the Underserved (MSATU), Dyrbye et al.^23^ inquired upon professional questions centered on the American Medical Association (AMA) code of ethics, and Brazeau et al.^24^ used the Professionalism Climate Instrument (PCI). Overall, medical students who experienced burnout were more likely to exhibit unprofessional behavior (Odds ratio 1.15–1.89; 35% to 21.9%).

### Mental health

The search and vetting process for burnout and relationship to mental health yielded 16 papers summarized in Table 3 (Appendix B). Professions studied included medical and dental students. Study designs included longitudinal, cross sectional cohorts and one systematic review. The MBI was the most frequently used survey (8 of 17 articles).^11^,^19^,^20^,^22^–^26^ Other surveys were used less frequently and catered to the needs of that particular study.^10^,^11^,^18^,^23^,^27^–^29^ Reed et al.^30^ noted that burnout correlated highly with depersonalization, stress, and emotional exhaustion in schools that used a grading scale system, which suggests pass/fail-grading systems may be beneficial to students’ mental health. Adding to this, Dyrbye et al.^23^ found that only 26.9% of medical students would definitely seek professional help for mental health problems, while 44.3% of the general population said that they would seek professional help for the same issues.

### Empathy

The effects of burnout on empathy in graduate healthcare students are summarized in Table 4 (Appendix B). Four of the six studies focused solely on medical students.^7^,^11^,^14^,^24^ However, in two of the six studies,^31^,^32^ HCP populations such as doctor of nursing practitioner, occupational therapy, physical therapy, and physician assistants were also examined along with medical doctor students. The MBI was utilized most frequently, while other assessment tools were utilized to evaluate students’ empathy. Four of the studies were cross-sectional cohorts.^7^,^11^,^14^,^24^,^32^ and one was a literature review.^33^ The cross-sectional studies demonstrated that empathy and compassion satisfaction exhibited an inverse relationship with depersonalization, emotional exhaustion, and burnout (p<0.001–0.02, OR=0.56–0.81). Positive correlations among personal achievement, altruism, and empathy were also documented in the cohorts (p<0.001). While the literature review noted the difficulty of incorporating humanism and empathy into graduate healthcare education, the articles investigating mindfulness, self-reflection, perspective taking, role modeling, and emotional labor are potential methods of increasing empathy, altruism, and prosocial behavior in graduate healthcare students.^33^

### Academic performance

Two studies, examining the effect of burnout on academic acuity, met the inclusion criteria (Table 5 in Appendix B). Grade point average (GPA), various scales of depression, engagement, and perfectionism were investigated to their correlation to academic success in medical and dental students. Atalayin et al.^34^ observed that 17.9% of students reported diminished academic acuity. Positive traits, such as self-oriented perfectionism, correlated with higher GPA’s and classroom engagement, whereas parental criticism was associated with burnout and depression. Seo et al.^35^ concluded that educational stressors were a significant factor in burnout.

## Discussion

In the scoping literature review on the effects of burnout in graduate healthcare students, 27 studies were identified that met the inclusion criteria. All studies included were published in the last ten years, in order to take into account the characteristics of the modern graduate healthcare student and contemporary medical education. Based on the aggregated surveys, we observed a high correlation between stress and burnout. Additionally, the scoping review indicates that graduate medical students display decreased life satisfaction compared to their age matched peers. We found an inverse relationship between burnout and empathy and professionalism. To further understand the most significant findings from this scoping review, we will discuss the consequences of high levels of stress and its effect on mental health and empathy.

Medical students experience higher levels of mental distress and depression than the general population and age-matched peers.^10^ Upon entering graduate programs, students do not differ significantly from their age matched peers. However, as students progress through their curricula, their mental health deteriorates and they experience higher levels of dissatisfaction.^29^ A decline in mental health can be mitigated via effective and healthy coping mechanisms.^20^ Healthy coping mechanisms often include physical activity, extracurricular activities, familiar and peer support networks, and professional counseling.^20^ Although many of these services are available in large academic institutions, they are often underutilized by the graduate student population due to perceived stigma.^23^ Because students in health professions are less likely to seek professional help, previous authors^10^,^30^ recommend that university programs stress the importance of healthy social networks, activities, and other coping mechanisms.^10^,^30^ Further research is needed to investigate the benefit and viability of various coping methods including exercise, mentorship, and free professional counseling services.

In addition to services offered to students with deteriorating mental health, strategies to mitigate decreased empathy,^14^,^17^ altruism,^11^ and compassion satisfaction^32^ have also been explored.^33^ Healthcare students experience burnout before they are exposed to treating patients.^17^ When students initiate treatment sessions with decreased empathy and compassion, poor patient–provider interactions and low quality of care result. Burks and Kobus^33^ proposed mindfulness, self-reflection, perspective taking, role modeling, and emotional labor as potential methods of increasing empathy, altruism, and prosocial behavior in healthcare students. Understanding what methods might be effective to decrease burnout, will not only optimize healthcare professional education, but also create empathetic and compassionate patient providers.

Several gaps in the literature were identified through this investigation. Over 90% of the studies selected were cohort studies. Medical students comprised 85% of the burnout investigations, with only 15% of studies investigated other graduate level healthcare professional students. There was only one randomized control trial that met the inclusion criteria. The limited student variety and study type warrants cautious generalization and begs further research.

Further research examining the efficacy of techniques to improve mental health and empathetic behavior, in addition to broadening the variety of graduate healthcare students will enhance administrators and program directors’ ability to create relevant and accessible coping mechanisms into curriculum.

### Limitations

This literature review was limited to only English speaking papers and journals. While there may be more publications in other languages, based on our inclusion criteria, there was a low probability of finding pertinent information. It is possible that relevant articles were omitted despite our search strategy. While our search was rigorous, we did not include all gray literature and duplicate papers. Lastly, publications that included practicing healthcare professionals were excluded. Some of these investigations included subjects that were graduate healthcare students. Although certain professional populations within these specific studies would have been relevant, the inclusion of practicing healthcare clinicians was beyond the scope of this investigation. Further investigation into burnout in all forms of healthcare education and training is warranted.

### Conclusion

The prevalence of burnout is high within graduate healthcare students, and that burnout can have a negative effect on the mental health, empathy, and professional conduct of students. Understanding the occurrence and effects of burnout within graduate healthcare programs will support faculty and administration to better educate healthcare clinicians via strategic curriculum planning. Administrators armed with this knowledge could then prepare students to not only understand and recognize burnout, but also afford students opportunities to decrease it. Further investigation is warranted into understanding the prevalence and effect burnout has upon other graduate healthcare students. Additionally, intervention strategies at all primary levels are needed to understand how to more effectively create healthy coping strategies for graduate healthcare students.

## Footnotes

† The term “graduate healthcare students” will be used throughout the paper to identify those students who are pursuing a doctoral healthcare degree.

## Appendix A

### Search Strategy

a. The following mesh term was used May, 2016 in PubMed, CINAHL, and ERIC: (“burnout, professional”[Major] OR burnout[ti] OR “stress, psychological”[Major] OR “Attitude of Health Personnel”[major] OR Depression[major] OR Anxiety[major] OR Mental Health[major]) AND (Students, Health Occupations[mesh] OR “medical students”[ti] OR Schools, Medical[major] OR “medical school”[ti] OR Education, Graduate[major]).

## Appendix B

**Table 1:** Etiology

Author name & date, Title, Journal name	Population	Exposure	Outcome	Type
Chang et al. 2012. Survey of the Prevalence of Burnout, Stress, Depression, and the Use of Supports by Medical Students at One School American Psychiatry	336 Students in the first 3 years of American medical school at one university.	Modified Maslach Burnout Inventory Human Services Survey (MBI-HSS); PRIME-MD depression screening survey; the Perceived Medical School Stress Scale; questions on demographics and helpful programs to cope with stress.	depressive symptoms were reported by 60% of respondents;	Cross Sectional Cohort
Dyrbye et al. 2006. Systematic Review of Depression, Anxiety, and Other Indicators of Psychological Distress Among US and Canadian Medical Students. Academy of Medicine	40 articles found on medical student psychological distress	peer-reviewed English-language studies published between January 1980 and May 2005: reporting on depression, anxiety, and burnout among U.S. and Canadian medical students; Searches used combinations of the Medical Subject Heading Terms: medical student and depression, depressive disorder major, depressive disorder, professional burnout, mental health, depersonalization, distress, anxiety, or emotional exhaustion.	no studies of burnout among medical students were identified; a high prevalence of depression and anxiety among medical students, with levels of overall psychological distress consistently higher than in the general population and age-matched peers by the later years of training.	Systematic Review
Dybye, et al. 2008. Burnout and suicidal ideation among U.S. medical students. Annals of Internal Medicine	4287 medical students at 7 medical schools, with students at 5 institutions studied longitudinally	prevalence of suicidal ideation in the past year; and its relationship to burnout, demographic characteristics, and quality of life	Burnout was reported by 49.6% (95% CI, 47.5% to 51.8%) of students, and 11.2% (CI, 9.9% to 12.6%) reported suicidal ideation within the past year. In a sensitivity analysis that assumed all nonresponders did not have suicidal ideation, the prevalence of suicidal ideation in the past 12 months would be 5.8%. In the longitudinal cohort, burnout (P < 0.001 for all domains), quality of life (P < 0.002 for each domain), and depressive symptoms (P < 0.001) at baseline predicted suicidal ideation over the following year; In multivariable analysis, burnout and low mental quality of life at baseline were independent predictors of suicidal ideation over the following year; Of the 370 students who met criteria for burnout in 2006, 99 (26.8%) recovered; Recovery from burnout was associated with markedly less suicidal ideation, which suggests that recovery from burnout decreased suicide risk.	Cross-sectional 2007 and longitudinal 2006 to 2007 cohort study.
Dyrbye 2010. Relationship between burnout and professional conduct and attitudes among US medical students. JAMA	2682/4400 [61%] Medical students at 7 Minnesotan medical schools. 48.6% female to 51.4% male; 56.3% aged 25–30	Maslach Burnout Inventory (MBI) and its correlation to depression; measured by the 2-item Primary Care Evaluation of Mental Disorders (PRIME-MD)	1354 of 2566 (52.8%) had burnout	Cross sectional cohort
Lapinski et al. 2015. Factors Modifying Burnout in Osteopathic Medical School Academic Psychiatry	1294 osteopathic medical students in America.	Maslach Burnout Inventory, the Patient Health Questionnaire, the Stressors and their impact scale, students’ sleeping and studying habits, and students’ extracurricular involvement	Burnout was present in 516 (39.9 %) osteopathic medical students, and 1006 (77.0 %) met criteria for depression; females were 1.5 times more likely to be burned out in comparison to males; for the burnout subscales, males had lower emotional exhaustion, slightly higher depersonalization, and lower personal accomplishment. Lesbian/gay/bisexual/asexual students were 2.62 times more likely to be burned out compared with heterosexual students; depression and academic, personal, and family stressors were all strongly linked to overall burnout; modifiable factors: average hours of sleep, average hours spent studying, and club involvement linked to burnout.	Cross Sectional Cohort
Santen et al, 2010 Burnout in medical students: examining the prevalence and associated factors	249 medical students	Maslach Burnout Inventory Human Services Survey (MBIHSS) (adapted) and scales of stressors, assessment of workload, relaxation, control, accomplishment, support systems, and demographics	A moderate or high degree of burnout was seen in 21% of the first year class, 41% of the second year class, 43% of the third year class, and 31% of the fourth year class (Pearson chi-square, *P* < 0.05); a high degree of burnout was demonstrated in 2% of the first year class, 15% of the second year class, 10% of the third year class, and 13% of the fourth year class (Pearson chi-square, *P* < 0.05).	Cross-sectional Cohort survey

**Table 2:** Professionalism

Author name & date, Title, Journal name	Population	Exposure	Outcome	Type
Brazeau CM, 2010. Relationship between medical student burnout, empathy, and professionalism climate. Academy of Medicine	71.8% (127/177) 4th year medical students at one medical school; 48% were women	Maslach Burnout Inventory (MBI) and its correlation to Professionalism Climate Instrument (PCI) and the Jefferson Scale of Physician Empathy (JSPE)	higher medical student burnout were associated with lower medical student empathy scores and with lower professionalism climate scores; all findings were at a *p*<.001	Cross sectional cohort
Dyrbye 2010. Relationship between burnout and professional conduct and attitudes among US medical students. JAMA	2682/4400 [61%] Medical students at 7 Minnesotan medical schools. 48.6% female to 51.4% male; 56.3% aged 25–30	Maslach Burnout Inventory (MBI) and its correlation to professionalism, measured by the Medical Students’ Attitudes Toward Providing Care for the Underserved (MSATU) instrument	1354 of 2566 (52.8%) had burnout; 14% (362/2531) had opinions dealing with professional behavior consistent with medical guidelines; students with burnout were more likely to enact unprofessional behavior: 35.0% vs 21.9%; odds ratio [OR], 1.89; 95% confidence interval [CI], 1.59–2.24); burnout was the only factor to associate with conducting unprofessional behavior: OR, 1.76; 95% CI, 1.45–2.13)	Cross Sectional Cohort
Dyrbye 2015. A national study of medical students’ attitudes toward self-prescribing and responsibility to report impaired colleagues. Academy of Medicine.	4,402 (35%) of 12,500 medical students from the national MD database Physician Masterfile (PMF) were contacted. 45.1% were male, median age was 25.	Maslach Burnout Inventory (MBI) and its correlation to professional questions that correlate with the AMA Code of Medical Ethics, and other professional questions relating to the Charter on Medical Professionalism	students with burnout were more likely to indulge in unprofessional behavior: ORs 1.15–1.51; less likely to agree to having a responsibility to colleagues OR 0.87	Cross sectional cohort study.
Dyrbye 2012. A multiinstitutional study exploring the impact of positive mental health on medical students’ professionalism in an era of high burnout. Academy of Medicine	2,682/4,400 (61%) Medical students at 7 Minnesotan medical schools. female (48.6% versus 45.1%) male younger than 25 (32.6% versus 25.9%), and white (78.4% versus 68.4%)	Maslach Burnout Inventory (MBI) and students’ personal beliefs on questions based on the Medical Students’ Attitudes toward Providing Care for the Underserved (MSATU) instrument	mental health improved, professional behavior improved 33/113 (29.2%), 426/1,120 (38.0%), and 718/1,391 (51.6%); languishing, moderate and flourishing mental health endorsed professional conduct	Cross sectional cohort

**Table 3:** Mental health PEOT

Author name & date, Title, Journal name	Population	Exposure	Outcome	Type
Montero-Marin et al. 2014b Perceived Stress Latent Factors and the Burnout Subtypes: A Structural Model in Dental Students PLoS ONE	314 Spanish dental students enrolled in two universities during the 2010–11 academic year	PSQ and Burnout Clinical Subtype Questionnaire Student Survey	strong associations among perceived stress factors and the burnout characteristics were observed, although a distinct pattern of relations was observed for each burnout subtype	Cross Sectional Cohort
Dahlin and Runeson. 2007. Burnout and psychiatric morbidity among medical students entering clinical training: a three year prospective questionnaire and interview-based study. BMC Medical Education.	113 Swedish medical students at one university followed from 1st to 3rd year (2001–2004)	Personality (HP5-i) and Performance-based self-esteem (PBSE-scale) were assessed at first year, study conditions (HESI), Burnout (OLBI), Depression (MDI) at 1st and 3rd years; diagnostic interviews (MINI) were used at 3rd year to assess psychiatric morbidity	high burnout was predicted by Impulsivity trait, Depressive symptoms at 1st year and Financial concerns at 1st year; when controlling for 3rd year study conditions, Impulsivity and concurrent Workload remained; 21 (27%) had a psychiatric diagnosis, 6 of the 21 sought help; unadjusted analyses showed that psychiatric morbidity was predicted by high Performance-based self-esteem, Disengagement and Depression at 1st year	Longitudinal cohort
Kjeldstadli et al. 2006. Life satisfaction and resilience in medical school – a six-year longitudinal, nationwide and comparative study BMC Medical Education	236 students from 4 Norwegian universities in 1993–1999	Likert Scale (7 point) using a global one item measure, 36 item Basic Character Inventory, Perceived Medical School Stress Inventory, and Ways of Coping Checklist	life satisfaction decreased during medical school; medical students were as satisfied as other students in the first year of study, but reported less satisfaction in their graduation year; medical students who sustained high levels of life satisfaction perceived medical school as interfering less with their social and personal life, and were less likely to use emotion focused coping, such as wishful thinking, than their peers	Longitudinal Cohort
Lapinski et al. 2015. Factors Modifying Burnout in Osteopathic Medical School Academic Psychiatry	1294 Osteopathic Medical Students in America	web-based survey to assess burnout and depression in osteopathic medical students: including Maslach Burnout Inventory, the Patient Health Questionnaire, the Stressors and their impact scale, students’ sleeping and studying habits, and students’ extracurricular involvement	burnout was present in 516 (39.9 %) osteopathic medical students, and 1006 (77.0 %) met criteria for depression; females were 1.5 times more likely to be burned out in comparison to males; Depression and academic, personal, and family stressors were all strongly linked to overall burnout; finally, for modifiable factors, average hours of sleep, average hours spent studying, and club involvement appeared to be linked to burnout	Cross Sectional Cohort
Van Venrooij et al. 2015. Burnout, depression and anxiety in preclinical medical students: a cross-sectional survey International Journal of Adolescent Medicine and Health	All preclinical medical students of Leiden University Medical Center. 433 responders	Maslach Burnout Inventory-General Survey (MBI-GS), depression and anxiety-related symptoms and vitality using the Symptom Questionnaire-48 (SQ-48); duration of sleep, quality of life (SF-36), need for recovery, happiness and dispositional optimism were assessed and analysed	prevalences of self-reported burnout-, depression- and anxiety-related symptoms were 46.0% (n=199), 27.0% (n=117) and 29.1% (n=126), respectively; independent correlates for burnout-related symptoms were <6 h sleep per night (p=0.02), low happiness (p<0.001) and a high need for recovery (p<0.001). Independent correlates for both depression-and anxiety-related symptoms were low optimism (p<0.001; p<0.001, respectively), low happiness (p<0.001; p=0.001, respectively) and a high need for recovery (p=0.03; p<0.001, respectively)	Cross sectional cohort
Chang et al. 2012. Survey of the Prevalence of Burnout, Stress, Depression, and the Use of Supports by Medical Students at One School American Psychiatry	336 Students in the first 3 years of American medical school at one university.	Modified Maslach Burnout Inventory Human Services Survey (MBI-HSS), the two-question PRIME-MD depression screening survey, the Perceived Medical School Stress Scale, along with questions on demographics and helpful programs to cope with stress	depressive symptoms were reported by 60% of respondents; most helpful coping mechanisms reported were social support from peers and faculty, counseling services, and extracurricular activities	Cross Sectional Cohort
Dyrbye et al. 2015. The Impact of Stigma and Personal Experiences on the Help-Seeking Behaviors of Medical Students With Burnout. Academic Medicine	873 medical students at 6 American universities in 2012	measured burnout, symptoms of depression, and quality of life using validated instruments and explored help-seeking behaviors, perceived stigma, personal experiences, and attitudes toward seeking mental health treatment	a third of respondents with burnout (154/454; 33.9%) sought help for an emotional/mental health problem in the last 12 months; a smaller percentage of respondents would definitely seek professional help for a serious emotional problem (235/872; 26.9%) than of the general population (44.3%) and age-matched individuals (38.8%)	Cross sectional cohort
Dyrbye et al. 2006. Systematic Review of Depression, Anxiety, and Other Indicators of Psychological Distress Among US and Canadian Medical Students. Academy of Medicine	40 articles found on medical student psychological distress	peer-reviewed English-language studies published between January 1980 and May 2005 reporting on depression, anxiety, and burnout among U.S. and Canadian medical students. Searches used combinations of the Medical Subject Heading terms medical student and depression, depressive disorder major, depressive disorder, professional burnout, mental health, depersonalization, distress, anxiety, or emotional exhaustion	no studies of burnout among medical students were identified; studies suggest a high prevalence of depression and anxiety among medical students, with levels of overall psychological distress consistently higher than in the general population and age-matched peers by the later years of training	Systematic Review
Dyrbye LN, 2013. A multi-institutional study exploring the impact of positive mental health on medical students’ professionalism in an era of high burnout. Academy of Medicine	4,400 medical students at seven U.S. medical schools in 2009; 2,682/4,400 (61%) responded	surveyed in 2009 to assess mental health (categorized as languishing, moderate, and flourishing) and burnout	prevalence of suicidal ideation (55/114 [48.2%], 281/1,128 [24.9%], and 127/1,409 [9.1%]) and serious thoughts of dropping out (15/114 [13.2%], 30/1,128 [2.7%], and 14/1,409 [1.0%]) decreased as mental health improved from languishing, moderate, and flourishing, respectively (all P < .0001); this relationship between personal experience and mental health persisted independent of burnout (all P < .001); as mental health improved, the prevalence of unprofessional behaviors (i.e., cheating and dishonest behaviors) also declined	Cross Sectional Cohort
Dybye, et al. 2008. Burnout and suicidal ideation among U.S. medical students. Annals of Internal Medicine	4287 medical students at 7 medical schools, with students at 5 institutions studied longitudinally	Survey to track prevalence of suicidal ideation in the past year and its relationship to burnout, demographic characteristics, and quality of life	burnout was reported by 49.6% (95% CI, 47.5% to 51.8%) of students, and 11.2% (CI, 9.9% to 12.6%) reported suicidal ideation within the past year; in a sensitivity analysis that assumed all nonresponders did not have suicidal ideation, the prevalence of suicidal ideation in the past 12 months would be 5.8%; in the longitudinal cohort, burnout (P < 0.001 for all domains), quality of life (P < 0.002 for each domain), and depressive symptoms (P < 0.001) at baseline predicted suicidal ideation over the following year; in multivariable analysis, burnout and low mental quality of life at baseline were independent predictors of suicidal ideation over the following year; Of the 370 students who met criteria for burnout in 2006, 99 (26.8%) recovered; recovery from burnout was associated with markedly less suicidal ideation, which suggests that recovery from burnout decreased suicide risk	Cross-sectional 2007 and longitudinal 2006 to 2007 cohort study.
Dahlin ME, Runeson B. 2007. Burnout and psychiatric morbidity among medical students entering clinical training: a three year prospective questionnaire and interview-based study. BMC Medial Education	127 first year medical students who were then followed-up at 3rd year of medical school	questionnaire to 127 first year med students and 81 follow up Interviews to 3rd year med students	98 (77%) responded on both occasions, 80 (63%) of these were interviewed; high burnout was predicted by Impulsivity trait, Depressive symptoms at 1st year and Financial concerns at 1st year; when controlling for 3rd year study conditions, Impulsivity and concurrent Workload remained; of the interviewed sample 21 (27%) had a psychiatric diagnosis, 6 of whom had sought help; unadjusted analyses showed that psychiatric morbidity was predicted by high Performance-based self-esteem, Disengagement and Depression at 1st year, only the later remained significant in the adjusted analysis	Cross Sectional and longitudinal
Dyrbye et al. 2010. Factors associated with resilience to and recovery from burnout: A prospective, multi-institutional study of US medical students. Medical Education	A total of 1321 medical students attending five institutions	Wilcoxon-Mann-Whitney test or Fisher’s exact test to evaluate burnout, quality of life, fatigue and stress	no differences in demographic characteristics were observed between resilient (290/792 [36.6%]) and vulnerable (502/792 [63.4%]) students; resilient students were less likely to experience depression, had a higher quality of life, were less likely to be employed, had experienced fewer stressful life events, reported higher levels of social support, perceived their learning climate more positively and experienced less stress and fatigue (all p < 0.05) than vulnerable students	Cross sectional Cohort
Dyrbye 2012, A multiinstitutional study exploring the impact of positive mental health on medical students’ professionalism in an era of high burnout. Academy of Medicine	2,682/4,400 (61%) Medical students at 7 minnesotan medical schools. female (48.6% versus 45.1%) male younger than 25 (32.6% versus 25.9%), and white (78.4% versus 68.4%)	Maslach Burnout Inventory (MBI), with 3 domains: emotional exhaustion (EE), depersonalization (DP), and low sense of personal accomplishment (PA) and its correlation to mental health by the Mental Health Continuum Short Form (MHC-SF), personal experiences including dropping out of MD school and suicidal ideation, and lastly students personal beliefs on questions based on the Medical Students’ Attitudes toward Providing Care for the Underserved (MSATU) instrument	prevalence of suicidal ideation (55/114 [48.2%], 281/1,128 [24.9%], and 127/1,409 [9.1%]) and serious thoughts of dropping out (15/114 [13.2%], 30/1,128 [2.7%], and 14/1,409 [1.0%]) decreased as mental health improved from languishing, moderate, and flourishing, respectively (all P < .0001)	Cross sectional cohort
Brazeau CM, 2010. Relationship between medical student burnout, empathy, and professionalism climate. Academy of Medicine	71.8% (127/177) 4th year medical students at one medical school 48% women,	Maslach Burnout Inventory (MBI) and its correlation to Professionalism Climate Instrument (PCI) and the Jefferson Scale of Physician Empathy (JSPE)	higher medical student burnout were associated with lower medical student empathy scores and with lower professionalism climate scores all at p<.001	Cross sectional cohort
Dyrbye 2010. Relationship between burnout and professional conduct and attitudes among US medical students. JAMA	2682/4400 [61%] Medical students at 7 minnesotan medical schools. 48.6% female to 51.4% male; 56.3% aged 25–30;	Maslach Burnout Inventory and it correlation to depression, measured by the 2-item Primary Care Evaluation of Mental Disorders (PRIME-MD), quality of life, measured by the Medical Outcomes Study Short-Form (SF-8), professionalism, measured by the Medical Students’ Attitudes Toward Providing Care for the Underserved (MSATU) instrument	1354 of 2566 (52.8%) had burnout; 14% (362/2531) had opinions dealing with professional behavior consistent with medical guidelines; students with burnout were more likely to enact unprofessional behavior: 35.0% vs 21.9%; odds ratio [OR], 1.89; 95% confidence interval [CI], 1.59–2.24	Cross sectional cohort
Dyrbye 2015. A national study of medical students’ attitudes toward self-prescribing and responsibility to report impaired colleagues. Academy of Medicine	4,402 (35%) of 12,500 medical students from the national MD database Physician Masterfile (PMF) were contacted. 45.1% were male, median age was 25.	Maslach Burnout Inventory (MBI) and its correlation to depression, measured by the two-item Primary Care Evaluation of Mental Disorders (PRIME-MD) instrument, alcohol abuse by the Alcohol Use Disorders Identification Test version C (AUDIT-C), professional questions that correlate with the AMA Code of Medical Ethics, and other professional questions correlating to the Charter on Medical Professionalism	students with burnout were more likely to indulge in unprofessional behavior: ORs 1.15–1.51	Cross sectional cohort

**Table 4:** Empathy PEOT

Author name & date, Title, Journal name	Population	Exposure	Outcome	Type
Brazeau CM, et al. 2010. Relationships between medical student burnout, empathy, and professionalism climate Academy of Medicine	127 4th-year medical students	Maslach Burnout Inventory (MBI), Jefferson Scale of Physician Empathy-Student (JSPES), Professionalism Climate Inventory (PCI)	emotional exhaustion and depersonalization, signs of burnout, were negatively associated with physician empathy; personal achievement was positively associated with physician empathy	Cross Sectional Cohort
Dyrbye LN, et al. 2010. Relationship between burnout and professional conduct and attitudes among US medical students JAMA	2682 medical students at 7 different medical schools	MBI, Primary Care Evaluation of Mental Disorders (PRIME-MD), Medical Students’ Attitudes Toward Providing Care for the Underserved (MSATU), MA Ethical Guidelines of Gifts to Physicians from Industry	point increase in MBI emotional exhaustion or depersonalization, or 1 point decrease in personal achievement, was associated with decreased likelihood of holding altruistic opinions about physicians’ responsibility to society; attitudes towards relationships with pharmaceutical industry inconsistent with AMA guidelines	Cross Sectional Cohort
Kulesa KC. 2014. Risk for Compassion Fatigue Among Doctor of Nursing Practice Students University of Arizona.	59 Doctor of Nurse Practitioner students at University of Arizona	Professional Quality of Life Scale-5 (ProQOL-5) - Screens for compassion fatigue risk in helping professionals (administered at beginning and end of training modules); participants learned about compassion fatigue and were given the option to complete the training or do other equivalent assignments	positive correlation between burnout and secondary traumatic stress; burnout and compassion satisfaction were negatively correlated; no relationship between compassion satisfaction and secondary traumatic stress	Cross Sectional Cohort
Barbosa P, et al.. 2013. Mindfulness-based stress reduction training is associated with greater empathy and reduced anxiety for graduate healthcare students Education Health (Abingdon)	13 experimental, 15 control, healthcare students (nursing, occupational therapy, physical therapy, physician assistant, and podiatric) at Samuel Merrit University (SMU)	administered mindfulness-based stress reduction (MBSR) training and investigated its effects on Burns Anxiety Inventory (BAI), Maslach Burnout Inventory (MBI), Jefferson Scale of Physician Empathy	BAI: 85% of experimental group experienced at least one level improvement at 8 and 11 weeks, 13% and 27% of control group at 8 and 11 weeks (respectively); SPE: experimental group significantly more empathetic at week 8, not at week 11 MBI: No significant differences -MBSR course evaluations: 4.7/5 ranking, 4.75/5 recommend	Randomized Control Trial
Burks DJ, Kobus AM.2012. The legacy of altruism in health care: the promotion of empathy, prosociality and humanism Medical Education	Concepts of altruism and prosocialism in healthcare; the prevalence of burnout; the impact of empathy on burnout, altruism, and prosocial behavior	N/A	humanism and empathy are difficult to incorporate and measure in health care provider (HCP) education; mindfulness, self-reflection, perspective taking, role modelling, and emotional labour are potential methods of increasing empathy, altruism, and prosocial behavior in HCPs	Literature Review

**Table 5:** Academics PEOT

Author name & date, Title, Journal name	Population	Exposure	Outcome	Type
Seo JH et al. 2015. Educational and Relational Stressors Associated with Burnout in Korean Medical Students Psychiatry Investigation	263 medical students attending Gyeongsang National University	standardized questionnaire survey using the Maslach Burnout Inventory was used to investigate educational and relational stressors, three dimensions of burnout, and social support of medical students; social support was measured by the 12-item Multidimensional Scale of Perceived Social Support	a significant proportion of students reported high levels of burnout: 28.1% for Emotional Exhaustion (EE) (≥19), 40.4% for Depersonalization (DP) (≥10), and 40.8% for Personal Accomplishment (PA) (≤14); 34% of the students reported two dimensions or more of high burnout; 16% of students suffered from both EE and DP, and 13.7% from both EE and PA, whereas 24.3% suffered from both DP and PA; students who reported three dimensions of high burnout, i.e., “totally burned out,” accounted for 9.9% of the total	Cross Sectional Cohort
Atalayin et al. 2015. The prevalence and consequences of burnout on a group of preclinical dental students. European Journal of Dentistry	329 preclinical dental students	Maslach Burnout Inventory student version, academic satisfaction scale, and personal information sheet were used to gather data	approximately 22.3% of students had high level of emotional exhaustion, 16.7% of students had high level of cynicism, and 17.9% of students suffered from high level of reduced academic efficacy; while the students attending the first year reported increased level of reduced academic efficacy, the students in the third year reported higher level of emotional exhaustion; academic workload played an important role in the development of burnout; students with high level of burnout reported less level of academic satisfaction and academic achievement	Cross sectional cohort
